# OA02.04. Integrative Health Coaching: the accumulating research at Duke IM

**DOI:** 10.1186/1472-6882-12-S1-O8

**Published:** 2012-06-12

**Authors:** J Kosey, L Simmons, A Perlman, L Smith, R Wolever

**Affiliations:** 1 Duke Integrative Medicine, Durham, USA

## Purpose

Lifestyle behaviors are the main contributor to chronic disease, including cardiovascular disease (CVD), diabetes, and obesity. However, few patients successfully make and sustain behavioral changes. To address this problem, Integrative Health Coaching (IHC) has emerged as a potential solution.

## Methods

Duke Integrative Medicine (IM) has completed 3 RCTs, a rigorous observational study, and a large program evaluation utilizing IHC.

## Results

In the first RCT, targeting CVD prevention, IHC improved 10 year CVD risk scores (Framingham) faster and more substantially than did usual care (UC). IHC patients also increased exercise and reduced their blood pressure, and the overweight IHC patients had greater weight loss. In a second RCT, patients with type 2 diabetes received 6 months of IHC. Compared to UC, the IHC group improved medication adherence, patient activation, exercise frequency, social support and benefit-finding. Those with baseline HbA1c > 7.0 also improved glycemic control. Subsequently, when the UC group also received IHC, additional improvements were captured including mood, perceived stress, and health-related quality of life. A third RCT examined the effects of mindfulness-based experiential education paired with IHC compared to an attention, education support control on weight loss maintenance. While both groups maintained significant weight loss eighteen months post-baseline, those in the IHC group lost additional weight. In a fourth study, a 3-day immersion with 8 months of IHC follow-up reduced 5 year stroke and diabetes risk through small improvements in multiple parameters (e.g., exercise behavior, resting pulse, BMI, waist circumference, and cholesterol). Finally, results from a health promotion program utilizing IHC showed a decline in inpatient admissions for those receiving IHC.

## Conclusion

Although in its infancy, IHC research demonstrates improved health outcomes and potentially reduced healthcare costs. While the health coaching field continues to evolve, the accumulating results of IHC merit a large-scale, multi-site RCT.

